# Challenges in Diagnosis and Clinical Management of COVID-19 in Patient with B-Cell Chronic Lymphocytic Leukemia (CLL): Report of One Case

**DOI:** 10.3390/hematolrep14010006

**Published:** 2022-03-17

**Authors:** Vincenzo Visco, Maria Enrichetta Lippi, Gerardo Salerno, Maria Angela Vittoria A. C. Licata, Chiara de Dominicis, Giusy Antolino, Giacinto La Verde, Iolanda Santino, Maurizio Simmaco, Salvatore Sciacchitano

**Affiliations:** 1 Department of Clinical and Molecular Medicine, Faculty of Medicine and Psychology, “Sapienza” University of Rome, 00185 Rome, Italy; gerardo.salerno@uniroma1.it (G.S.); gantolino@ospedalesantandrea.it (G.A.); giacinto.laverde@uniroma1.it (G.L.V.); salvatore.sciacchitano@uniroma1.it (S.S.); 2 Department of Clinical and Molecular Medicine, Sant’Andrea University Hospital, 00189 Rome, Italy; iolanda.santino@uniroma1.it (I.S.); maurizio.simmaco@uniroma1.it (M.S.); 3 Department of Cardiological Rehabilitation, IRCCS San Raffaele, 00163 Rome, Italy; mlippi@hotmail.it; 4 Pulmonary Diseases Unit, Department of Medical and Surgical Sciences, Fondazione Policlinico Universitario Agostino Gemelli, IRCCS, 00168 Rome, Italy; mav.licata@gmail.com; 5 Department of Surgical and Medical Sciences and Translational Medicine, “Sapienza” University of Rome, 00185 Rome, Italy; chiara.dedominicis@uniroma1.it; 6 Department of Neuroscience, Mental Health, and Sensory Organs (NESMOS), “Sapienza” University of Rome, 00185 Rome, Italy; 7 Laboratory of Biomedical Research, Niccolò Cusano University Foundation, 00166 Rome, Italy

**Keywords:** CLL, CLL infection, antibodies, molecular diagnosis, virology

## Abstract

We report here a case of a patient affected by B-cell chronic lymphocytic leukemia (CLL) that developed COVID-19 during the actual SARS-CoV-2 outbreak. The coexistence of CLL and COVID-19 raises many questions regarding the possible increased risk of developing COVID-19 among patients with CLL, the problems in managing therapies for both diseases and, above all, the difficulties in diagnosing COVID-19 in patients affected by CLL. In our patient, an 84-year-old man, the recognition of COVID-19 was delayed because of its atypical clinical presentation and technical problems related to the methods used for the diagnosis. Based on the symptoms and the radiological aspect of the lung, the occurrence of COVID-19 was suspected. Repeated tests on oro/nasopharyngeal swabs gave negative results, causing a delay in the diagnosis. Moreover, different methods used to identify the SARS-CoV-2 antibodies in serum gave conflicting results, and only two tests were able to identify SARS-CoV-2 Abs of the IgG type. During the clinical course of unrecognized COVID-19, our patient developed severe complications and did not receive any specific treatment for the two diseases. Recognition of COVID-19 in patients with CLL is a challenging task and the most accurate methods are necessary to overcome the diagnostic difficulties encountered.

## 1. Case Report

An 84-year-old obese man was diagnosed with CLL in 2006. In 2017, he underwent transcatheter aortic valve implantation (TAVI) because of the worsening of his atrial fibrillation due to aortic stenosis. Bone marrow aspiration, flow cytometry immunophenotyping and hematological investigations were performed over the course of disease, including a Binet stage C, Rai stage IV and a European Cooperative Oncology Group (ECOG) performance score of 1. The CLL was treated for several years only with prednisone at 10 mg per day.

In February 2020, based on a peak of lymphocytosis and flow cytometry analysis, treatment with ibrutinib was considered. However, he never started such treatment because in March 2020, he became symptomatic with a sore throat, fever (39 °C), dyspnea and a dry cough, associated with a paO2 < 80%. For this reason, he was hospitalized in the intensive care unit. On admission, he showed acute hypoxemic respiratory failure, with a pO2 = 35, and started non-invasive ventilation (NIV) therapy 12-10, with FiO2 55%.

**Figure 1:**
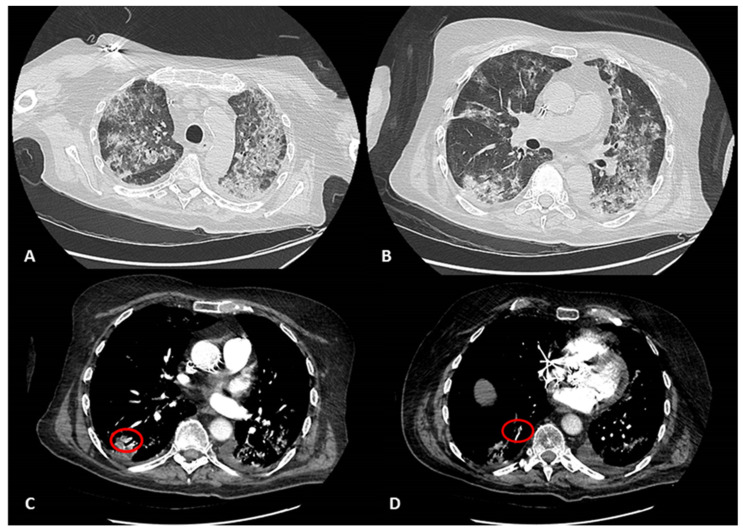
(**A**,**B**) CT scan of the chest showing multiple large bilateral patchy ground-glass opacities (GGOs) and consolidations, mostly involving peripheral lung parenchyma. (**C**,**D**) CT scan of the chest with iodinated contrast. Red circles: Thromboembolic alterations involving sub-segmentary pulmonary arteries.

High-resolution CT (HRCT) of the lung showed the typical aspect of interstitial pneumonia, with a “ground glass” appearance. The total severity score (TSS) and the COVID RADS were calculated in January 2020, before the onset of the symptoms, and in March 2020 during the acute phase of the disease according to the methods previously described [1,2]. TSS results were 0 and 16, and RADS scores were 0 and 4, respectively. The HRCT scan also showed signs of distal, bilateral lung thromboembolism (Figure 1), and treatment with fondaparinux (Arixtra) at 1.5 mg/day was administered. Based on the results of the HRCT scan and thromboembolic complications, considering the TSS and CO-RADS scores, the occurrence of SARS-CoV-2 infection was suspected, and nasal/oropharyngeal swabs followed by a real-time reverse-transcription polymerase chain reaction (rRT-PCR) test were performed. We used two different kits, namely the Allplex™ 2019-nCoV Assay (Seegene MuDT^TM^, Seoul, Republic of Korea) and the RNA Detection kit (DAAN Gene Co. LTD, Guangzhou, Guangdong, China). The result was negative with both. In agreement with WHO recommendations in case of suspected cases, we repeated the test many times, with the same negative results (Table 1). Therefore, the patient was not transferred to the isolation ward for appropriate patient management in accordance with the specific COVID-19 Integrated Care Pathway (ICP), recently published [3]. He was admitted first to intensive care and then the pneumatological unit, where a bronchoalveolar lavage (BAL) was performed without any special protection. Physicians attending to him were therefore potentially exposed to infection. The BAL specimen was subjected to cytological assessment and culture, in search of other possible bacterial and fungal infections. On that occasion, another attempt was made to detect the SARS-CoV-2 virus in the BAL specimen using the kit by Seegene. The rRT-PCR failed to identify the virus, probably because of the delayed timing of the exam.

During the acute phase of the disease, our patient did not show the typical laboratory findings of a COVID-19 patient, consisting of marked lymphocytopenia and a marked reduction in both CD8+ and CD4+ T cells. The laboratory test, on the contrary, showed marked lymphocytosis and thrombocytopenia, reported in Table 1. The lymphocytosis observed in our patient with CLL was responsible for the masking of the occurrence of COVID-19 [4,5]. Nevertheless, during hospitalization, the clinical presentation and the radiological aspect of the lung strongly suggested the presence of SARS-CoV-2 infection. We therefore requested the identification of specific immunoglobulins in serum directed against the SARS-CoV-2 virus using different kits (EUROIMMUN Anti-SARS-CoV-2 Ig ELISA; Abbott SARS-CoV-2 Ig assay; Maglumi™ 2019-n-Cov: IgG and IgM automated quantitative chemiluminescent immunoassays (CLIA); Human Anti-2019 nCoV(S) Ig by ELISA Fine Test-Wuhan Fine Biotech Co., Ltd., Wuhan, China). IgM of SARS-CoV-2 always resulted negative, whereas a repeated search of IgG using the different kits gave contradictory results. Only two assays (Maglumi and Fine Test) were able to identify the presence of SARS-CoV-2 IgG antibodies. Therefore, in addition to the difficulties in identifying the disease based on the laboratory results, we had to face the difficulties related to the specificity and sensitivity of the different tests recommended for the diagnosis of COVID-19.

In our patient, the clinical course of COVID-19 was severe. While at the intensive care unit, he received non-invasive ventilatory (NIV) therapy until the dyspnea subsided. He also developed a thromboembolic complication and an *Acinetobacter baumanii* infection of the lung. He did not receive any treatment for COVID-19, and he could not be treated with ibrutinib for CLL as well. The patient is now in good clinical condition, with some respiratory complications but a globally well-preserved pulmonary function.

## 2. Ethical Approval

Written informed consent was obtained from the participants of the study. The study was approved by our Institutional Ethical Committee (University Sapienza of Rome, Italy) (Prot.# 52SA_2020, RIF. CE5773_2020), on the basis that it complied with the Declaration of Helsinki and that the protocol followed existing good clinical practice guidelines.

## 3. Discussion and Conclusions

The clinical management of this patient raised several points that deserve discussion. The first point concerns the diagnosis of COVID-19 and the accuracy of the currently available tests. It appears that the detection of SARS-CoV-2 in nasal/oropharyngeal swabs, considered the reference standard for COVID-19 diagnostics, is not so accurate and sensitive in all cases. Repeated tests in our patient constantly gave negative results. In other similar cases of coexisting CLL and COVID-19, real-time RT-PCR was able to detect the SARS-CoV-2 virus [4]. This was not the case in our patient. According to WHO guidelines (Laboratory testing for coronavirus disease (COVID-19) in suspected human cases, 2020) [6] and based on the protocol developed by Charitè, Berlin, Germany [7], the diagnosis should be based on the demonstration of the virus in nasal/oropharyngeal swabs, analyzed by rRT-PCR. In severe cases, the most suitable samples, showing a higher RNA-positive rate, seem to be BAL or deep sputum [8]. The two most frequently used and recommended kits, both CE-approved, are the one by Seegene (Allplex™ 2019-nCoV Assay) and the one by DAAN (RNA Detection kit). The first one is based on the identification of at least two out of the three genes considered (E, RdRP and N genes), while the second one is based on the identification of only one of the two genes examined (ORF1ab and N genes). In our patient, both kits repeatedly failed to demonstrate the presence of the virus in the nasal/oropharyngeal swabs and the BAL too. Therefore, the initial diagnosis was delayed because of the inability of these two kits to detect the virus. It has been previously reported that molecular tests carried out through swabs can give false negative results in people who harbor the virus. This happens because swab collection and time of sampling are critical. This could be a possible explanation of the false negative results at least in the last test, which was performed too late (weeks later) when the virus could no longer be present in the BAL specimen. Although such repeated negative results may be related to this specific patient, they could reflect a lack of sensitivity in the panels used for RT-PCR [9,10]. However, as suggested by Niu A et al. [9], since patients with hematological diseases have a high risk of developing a severe form of COVID-19 and are also more susceptible to false negative RT-PCR tests, the diagnostic and therapeutic management of this selected population should be more aggressively performed.

We were able to make the diagnosis only after the acute phase, when there was a rise in the specific IgG. The presence of specific IgG directed against the viral antigens was demonstrated, in fact, only after discharge from the hospital. Serology tests are useful in identifying individuals who may have developed an immune response to SARS-CoV-2. Furthermore, it has been suggested that they may be of great help to overcome lockdowns [11] and to allow a population-wide estimation of infected people. However, they are not considered useful for diagnosing COVID-19, mostly because they cannot be detected in the early days of infection. They may be useful if the diagnosis is missed at admission and can only be obtained later during recovery.

The second point concerns the accuracy of the different commercially available methods to detect the immunoglobulins. The sensitivity of the methods used to detect specific immunoglobulins in serum, in fact, varies according to the method used. The method that, in our experience, was able to detect specific IgG in the serum was the one that is directed toward two different antigens, namely the nucleocapsid (N) protein and the spike (S) protein of the virus. The other methods we used, based on the detection of specific IgG directed toward only the N protein, gave a negative result. This is in agreement with previous observations regarding the accuracy of these methods [12,13]. In many countries, the use of these tests in the general population has been planned to obtain epidemiological information regarding the diffusion of the virus in order to identify and isolate persons who are contagious and to establish the real lethality rate of the disease. We should consider that the World Health Organization (WHO) recently stated that there is not enough evidence about the effectiveness of antibody-mediated immunity to guarantee the accuracy of an ‘immunity passport’ or ‘risk-free certificate’ (World Health Organization. “Immunity passports” in the context of COVID-19 [14]. In any case, considering the potential pitfalls and benefits of such tests as well as the ethical and economic issues [15], the use of serological antibody testing is rapidly emerging [16].

In Italy, an epidemiological study is currently ongoing for the detection of anti-SARS-CoV-2 N antigen-specific IgG in a representative group of 150,000 people [17]. It would be extremely relevant if such information could be based on the most accurate test available. An exhaustive list of all the kits has recently been published by the FDA [18]. They can be divided into those that recognize only one single target (the nucleocapsid, the spike protein or the receptor-binding domain) and those that recognize more than one target.

In this regard, a test that considers different epitopes in two separate proteins of the same virus seems to be more sensitive and more appropriate to be used as a screening test. In our single patient experience, this kit was the only one that gave a positive result. It was more sensitive compared to the other similar kits and even more sensitive compared to the rRT-PCR-based methods.

The third point concerns the occurrence of lymphocytosis. In our patient, we observed a marked rise in the serum concentration of lymphocytes that was presumably coincident with the onset of COVID-19. It was interpreted as a secondary effect of corticosteroid therapy, and this could be one of the plausible explanations. This finding has been previously reported in other therapy-naive CLL patients with COVID-19 [5]. We should consider that lymphopenia is commonly present in patients with COVID-19. However, an opposite behavior of lymphocytes in patients affected by CLL could be remarked. In fact, although only occasionally found, marked lymphocytosis in COVID-19 patients has been described, especially in those also affected by CLL [19]. This peak of lymphocytosis might partially mask the occurrence of COVID-19 (as previously suggested [4,5]), which was the case in our patient. However, we cannot exclude that the lymphocytosis may be related to the immune response to the viral infection.

The fourth point is the specificity of the clinical presentation and of the radiological images. Both the clinical presentation and the radiological appearance at the HRCT scan of the lung were highly suggestive of the occurrence of COVID-19 in our patient. They represent, in fact, the reason why we kept trying to identify the disease and to reach the diagnosis in our patient.

In conclusion, in CLL patients showing a clinical presentation and radiological appearance of the lung suggesting the possible coexistence of COVID-19, the confounding hematological factors that may mask the disease and limit the diagnosis of SARS-CoV-2 infections should be taken into careful consideration.

All possible attempts should be made to obtain the correct identification of the disease, using the most accurate methods, to avoid misdiagnosis and delay in identifying the infection, thus preventing the spreading of the virus.

## Figures and Tables

**Table 1:** The diagnostic procedures (RT-PCR, SARS-CoV-2 IgG, blood cell counts, symptoms) and medical history are summarized.

	FEBRUARY										MARCH											APRIL										MAY								
RT-PCR-SARS CoV-2																	**-**		**-**	**-**					**-**															
SARS CoV-2 IgG α -N ^(a)^																														**-**										
SARS CoV-2 IgG α -N ^(b)^																																								**-**
SARS CoV-2 IgG α -N+S																																								**+**
SARS CoV-2 IgG α-S																																								**+**
																																								
Blood WBC Count *	**17**					**47**					**62**		**61**		**45**				**42**		**35**								**41**		**25**		**13**							
Blood Lympho Count *	**13**					**40**					**54**		**49**		**37**				**36**		**24**								**38**		**22**		**11**							
Blood Neutro Count *	**2, 6**					**6**					**7, 2**		**6, 2**		**4, 8**				**4, 6**		**4, 1**								**2, 3**		**1, 8**		**1, 6**							
																																								
Fever > 37.5°										**+**	**+**																													
Dyspnea																**+**	**+**	**+**	**+**	**+**	**+**	**+**	**+**	**+**	**+**	**+**	**+**	**+**	**+**	**+**	**+**	**+**	**+**	**+**	**+**	**+**				
Cough													**+**	**+**	**+**	**+**	**+**	**+**	**+**	**+**	**+**	**+**	**+**	**+**	**+**	**+**	**+**	**+**	**+**	**+**	**+**	**+**	**+**	**+**	**+**	**+**	**+**	**+**	**+**	**+**
																																								
Emergency Room																																								
Intensive Care Unit																																								
Pneumology																																								
Hospitalization																																								

*: ×10^9/L^; (a): EUROIMMUN; (b): Abbott.
